# Clinical outcomes of IgA nephropathy patients with different proportions of crescents

**DOI:** 10.1097/MD.0000000000006190

**Published:** 2017-03-24

**Authors:** Wang Zhang, Qian Zhou, Lingyao Hong, Wenfang Chen, Shicong Yang, Qiongqiong Yang, Wei Chen, Xueqing Yu

**Affiliations:** a Department of Nephrology, The First Affiliated Hospital, Sun Yat-sen University, Key Laboratory of Nephrology, Ministry of Health; b Department of Pathology, The First Affiliated Hospital, Sun Yat-sen University, Guangzhou, China.

**Keywords:** crescent, IgA nephropathy, pathology, prognosis

## Abstract

Crescents involving more than 50% of glomeruli in IgA nephropathy (IgAN) signify a rapid deterioration of renal function. However, little is known about the prognosis of IgAN patients presenting crescents in less than 50% of glomeruli. We aimed to investigate the clinicopathological characteristics and outcomes of IgAN patients with different proportions of crescents.

From January 2000 to December 2011, biopsy-proven primary IgAN patients with histological crescents formation were enrolled in this retrospective cohort study. The patients were divided into 4 groups on the basis of crescent proportion as follows: <5%, 5% to 9%, 10% to 24%, and ≥25%. The primary endpoint was defined as the doubling of baseline serum creatinine (SCr) and/or end-stage renal disease (ESRD), and the secondary endpoint was death.

A total of 538 crescent-featured IgAN patients were followed up and included in the analysis. The median crescent proportion was 8.0%. An increasing crescent proportion was associated with a reduced estimated glomerular filtration rate (eGFR), decreased level of hemoglobin, and increased amount of urine protein excretion. After a median follow-up period of 51 months (range 12–154 months), the endpoint events-free survival rate of the above 4 groups were 69.9%, 47.7%, 43.8%, and 40.6%, respectively (Log rank=13.7, *P*= 0.003), when we incorporated death with renal outcome as a composite endpoint. Multivariate Cox regression analyses adjusting for eGFR, hypertension, proteinuria, and the Oxford-MEST classification demonstrated the predictive significance of an increasing crescent proportion with renal survival and mortality (each increase by 5% [log-transformed]: HR=1.51, 95% CI 1.08–2.11, *P* = 0.02). Further comparisons of patients with small proportions of crescents (<5%) and those absent of such pathological lesion showed that the 2 groups of patients had comparable prognosis.

An increasing crescent proportion was identified as an independent predictor for unfavorable clinical outcomes in IgAN. Therefore, a small proportion of crescents, over 5% particularly, should be paid more attention in clinical practice.

## Abstract 2

Supplemental Digital Content is available in the text

## 1 Introduction

IgA nephropathy (IgAN) is the most common type of primary glomerulonephritis worldwide, which exhibits a widely varying clinical course from asymptomatic urinary abnormalities to rapidly progressive renal failure. A previous literature review showed that the prevalence of extracapillary proliferation in IgAN, usually characterized by noncircumferential crescents, ranges between 20% and 60%.^[1–6]^ The 2012 KDIGO Clinical Practice Guideline for Glomerulonephritis defined crescentic IgAN as IgAN with crescents involving more than 50% of glomeruli in the renal biopsy, which has been assumed to render rapidly progressive renal deterioration and poor outcomes. However, the prognosis of IgAN patients with crescents involving <50% glomeruli varies significantly.^[1–3,5–11]^ Therefore, we performed this retrospective cohort study to investigate the outcomes and associated risk factors for disease progression in IgAN patients with different proportions of crescents.

## 2 Methods

### 2.1 Study population

Patients who had renal biopsy-proven primary IgAN with histological crescent formation and were registered in the IgAN Database of The First Affiliated Hospital of Sun Yat-sen University (http://igan.medidata.cn) from January 2000 to December 2011 were retrospectively reviewed. The number of glomeruli was required to be at least 10 per biopsy section. Patients with age <14 years old, or secondary mesangial IgA deposition (systemic lupus erythematosus, Henoch–Schönlein purpura, hepatic diseases or lymphoma, etc.) or end-stage renal disease (ESRD) status on admission (eGFR <15 mL/min per 1.73 m^2^, maintenance hemodialysis, maintenance peritoneal dialysis, or renal transplantation), or without complete follow-up data were excluded. This study was carried out in accordance with the Declaration of Helsinki, and the study was approved by the Institutional Review Board (IRB) of the SYSU Clinical Trial Centre. All study subjects signed informed consent forms.

### 2.2 Clinical and pathological data evaluation

Baseline clinical and laboratory data, for example, eGFR and urine protein, were obtained at the time of renal biopsy from a review of medical records. The eGFR was calculated using an abbreviated Modification of Diet in Renal Disease (MDRD) equation modified for the Chinese adults: eGFR (mL/min per 1.73 m^2^) = 186 × PCr^−1.154^ × age^−0.203^ × .742 (if female) × 1.233.^[12]^ For adolescent patients aged 14 to 18 years, the Schwartz formula^[13]^ was introduced: eGFR = *k* × height / Cr (for girls: *k* = 48.6; for boys: *k* = 61.9). Hypertension was defined as systolic blood pressure ≥140 mm Hg and/or diastolic blood pressure ≥90 mm Hg or treatment with antihypertensive drugs. Anemia was defined by gender-specific criteria of hemoglobin concentrations <120 g/L in males and <110 g/L in females. Hyperuricemia was defined by gender-specific criteria of serum uric acid (UA) >420 μmol/L in males and >360 μmol/L in females. Hypercholesterolemia was defined as a total cholesterol level ≥5.2 mmol/L, and hypertriglyceridemia was defined as a total triglyceride level ≥1.7 mmol/L. Urine protein excretion was calculated from a 24-hour urine collection. The use of RAS (renin-angiotensin system) inhibitors indicated exposure to angiotensin-converting enzyme inhibitors (ACEI), angiotensin-receptor blockers (ARB), or both. Immunosuppressive therapy referred to receiving corticosteroids with or without cytotoxic agents.

Renal histopathological data were reviewed and re-classified by 3 experienced renal pathologists according to the Oxford classification criteria of IgA nephropathy.^[14]^ A crescent was defined as an extracapillary lesion of more than 2 cell layers involving >10% of the circumference of Bowman's capsule. A cellular crescent was >50% of the extracapillary lesion occupied by cells, and a fibrocellular crescent was an extracapillary lesion comprising cells and extracellular matrix with <50% cells and <90% matrix. A fibrous crescent was defined as >10% of the circumference of Bowman's capsule covered by a lesion composed of ≥90% matrix. The number of globally sclerotic glomeruli was counted in the total number of glomeruli. The proportion of crescents (regardless of the composition) was calculated as the number of crescent-affected glomeruli divided by the total number of glomeruli. Cellular/fibrocellular/fibrous crescents were calculated according to the relative ratio. Global and segmental glomerulosclerosis were calculated as the percentage of involved glomeruli. Interstitial inflammatory lesions, interstitial fibrosis, and tubular atrophy were evaluated semi-quantitatively on the basis of the affected cortical area: <25% = mild, 25–49% = moderate, and ≥50% = severe.

### 2.3 Clinical outcome

The primary endpoint was renal outcome comprising doubling of baseline serum creatinine (SCr) and ESRD (maintenance hemodialysis, maintenance peritoneal dialysis, or renal transplantation). The secondary endpoint was death.

### 2.4 Statistical analysis

Normally distributed variables were expressed as the mean± SD and compared using a *t* test or analysis of variance (ANOVA), as required. Nonparametric variables were expressed as the median (interquartile range, IQR) and compared using a Mann–Whitney *U* test or Kruskal–Wallis test. Categorical variables were expressed in frequencies (percentages) and compared using the chi-squared test. The cumulative survival rates were presented in Kaplan–Meier curves, and comparisons of survival were based on the log-rank test. The Cox proportional hazard regression model was used to assess the association of baseline variables with the clinical outcomes. To identify independent predictors of progression, we performed a multivariate Cox regression analysis with a selection of variables. Because the proteinuria and crescent proportion distributions were skewed, the log-transformed values were used in the regression analysis, and the significance was obtained with nontransformed data. Data were analyzed using SPSS 13.0 software (SPSS, Chicago, IL). A *P*-value <0.05 was considered statistically significant. All tests were 2-tailed.

## 3 Results

### 3.1 Baseline clinical and pathological characteristics

**Figure 1:**
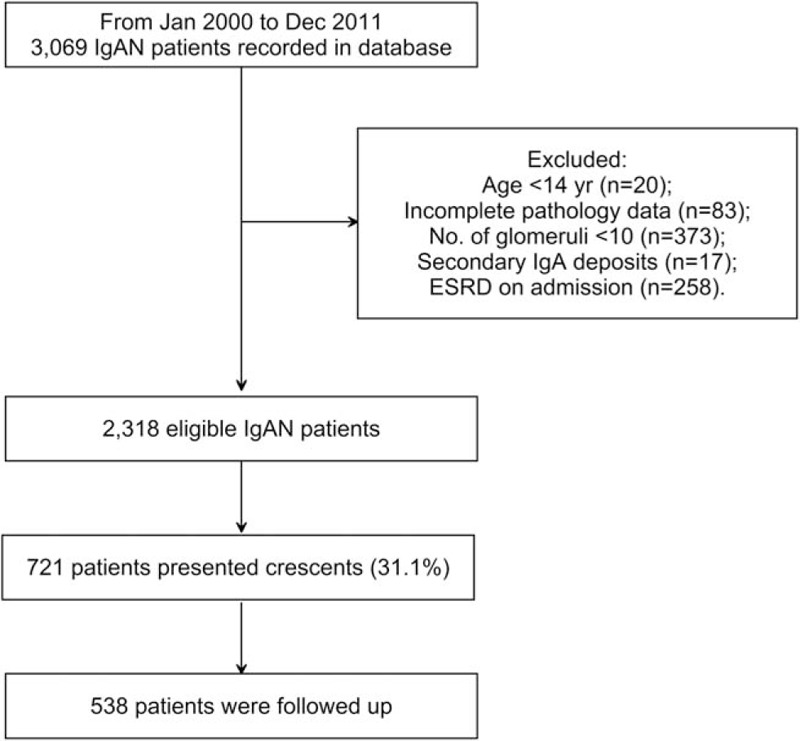
A flow diagram of the enrolment of IgAN patients with crescents. IgAN = IgA nephropathy.

From January 2000 until December 2011, a total of 2318 eligible IgAN patients were recorded in the database, 721 (31.1%) of whom presented crescents on biopsy (Fig. 1). Among crescent-featured individuals, 538 patients were followed up, whose baseline conditions were almost comparable to those lost to follow-up (see Table 1, Supplemental Content, which illustrates the comparisons of patients who were followed up or not). The 538 IgAN patients presenting crescents were further divided into 4 groups on the basis of crescent proportions: <5%, 5–9%, 10–24%, and ≥25% (Table 1). The median crescent proportion was 8.0% (IQR: 4.5–14.3%), including 6 cases of crescentic IgAN. A higher crescent proportion was associated with a lower eGFR, decreased hemoglobin levels, and increased amounts of urine protein excretion (all *P*-trend < 0.05). Moreover, a growing number of patients were administered immunosuppressive therapy, especially for the ≥25% group, in which approximately 70% of patients received oral corticosteroids and 39.6% received intravenous methylprednisolone pulse administration. In terms of pathological lesions, the crescent component (cellular or fibrous) was balanced among the groups, whereas the degrees of glomerulosclerosis, mesangial hypercellularity, endocapillary hypercellularity, and tubulointerstitial lesions were significantly different.

**Table 1:**
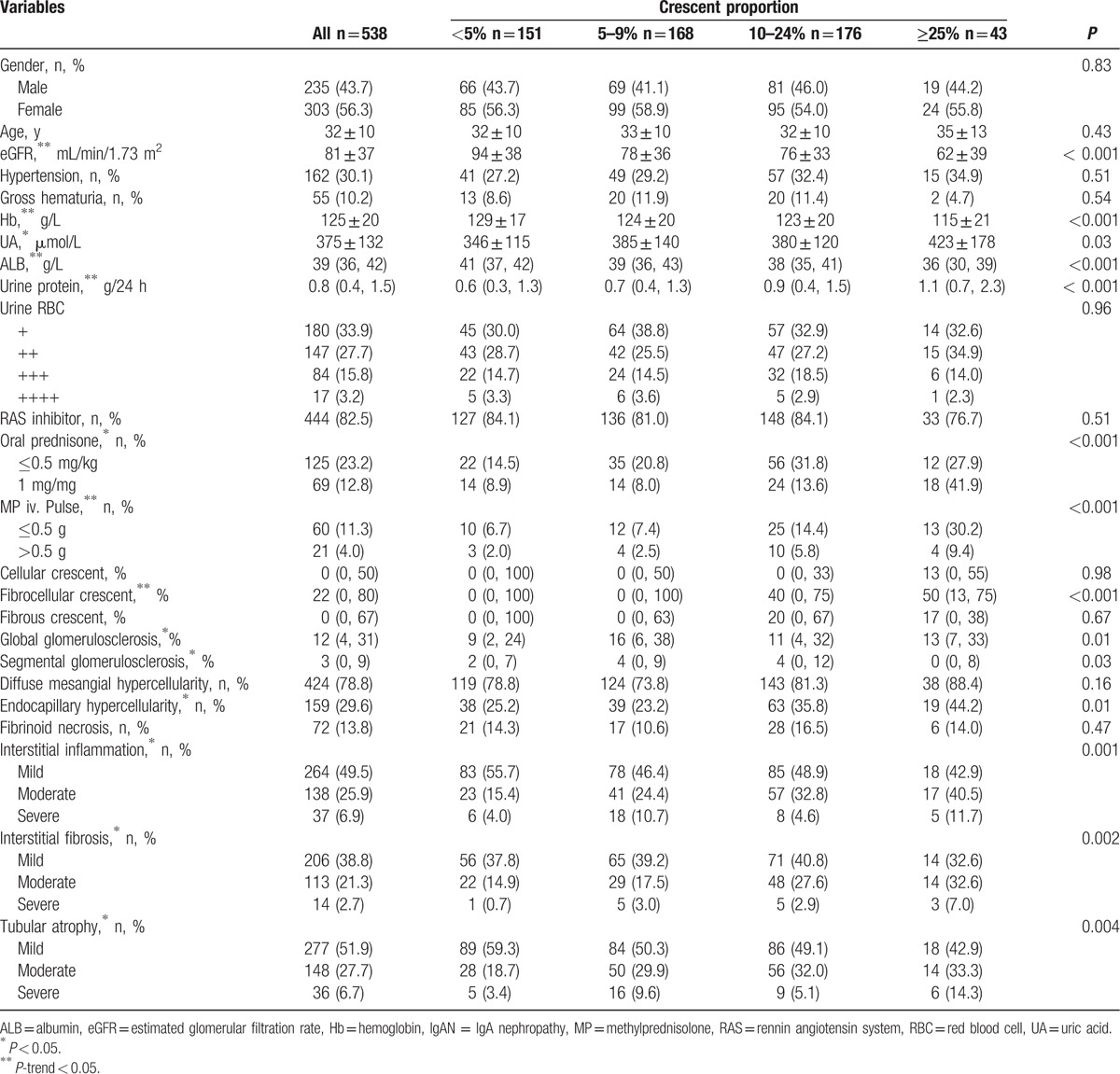
Baseline clinicopathological characteristics of IgAN patients in different proportions of crescents.

### 3.2 Predictive assessment of clinical outcomes

**Figure 2:**
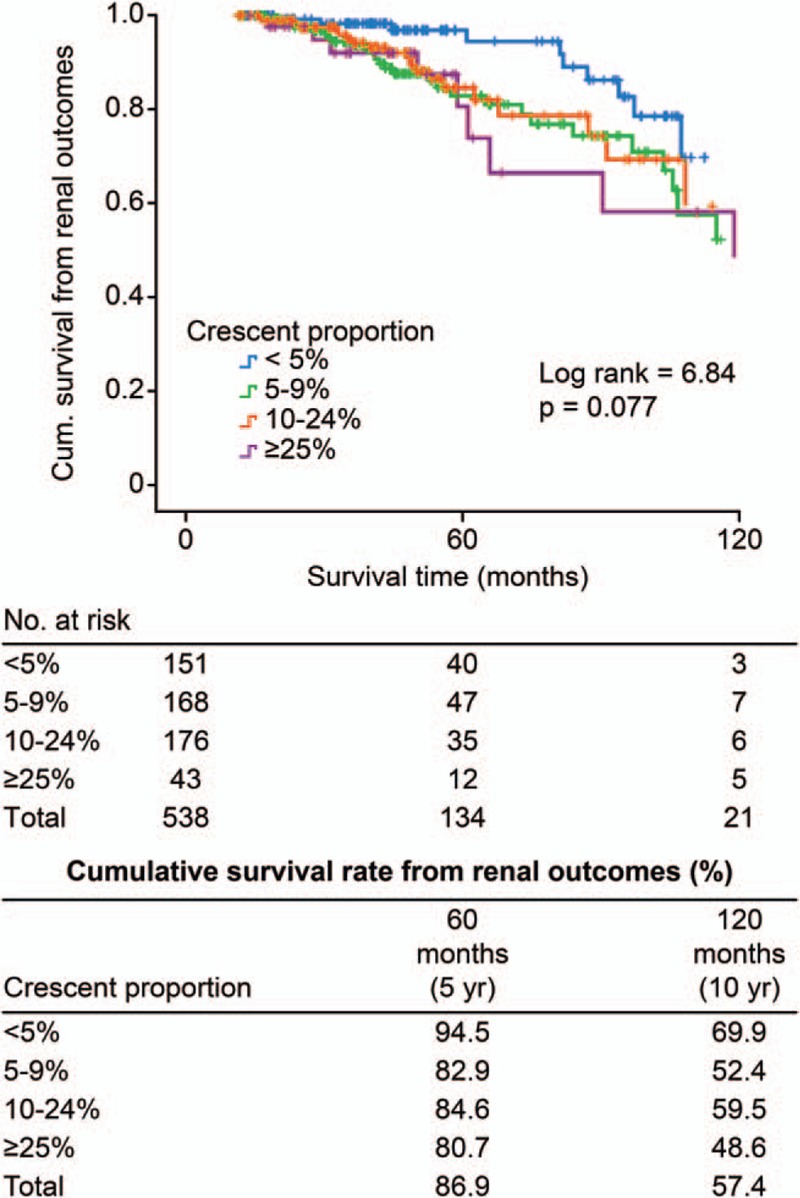
Renal outcomes in IgAN patients with different proportions of crescents. IgAN = IgA nephropathy.

**Figure 3:**
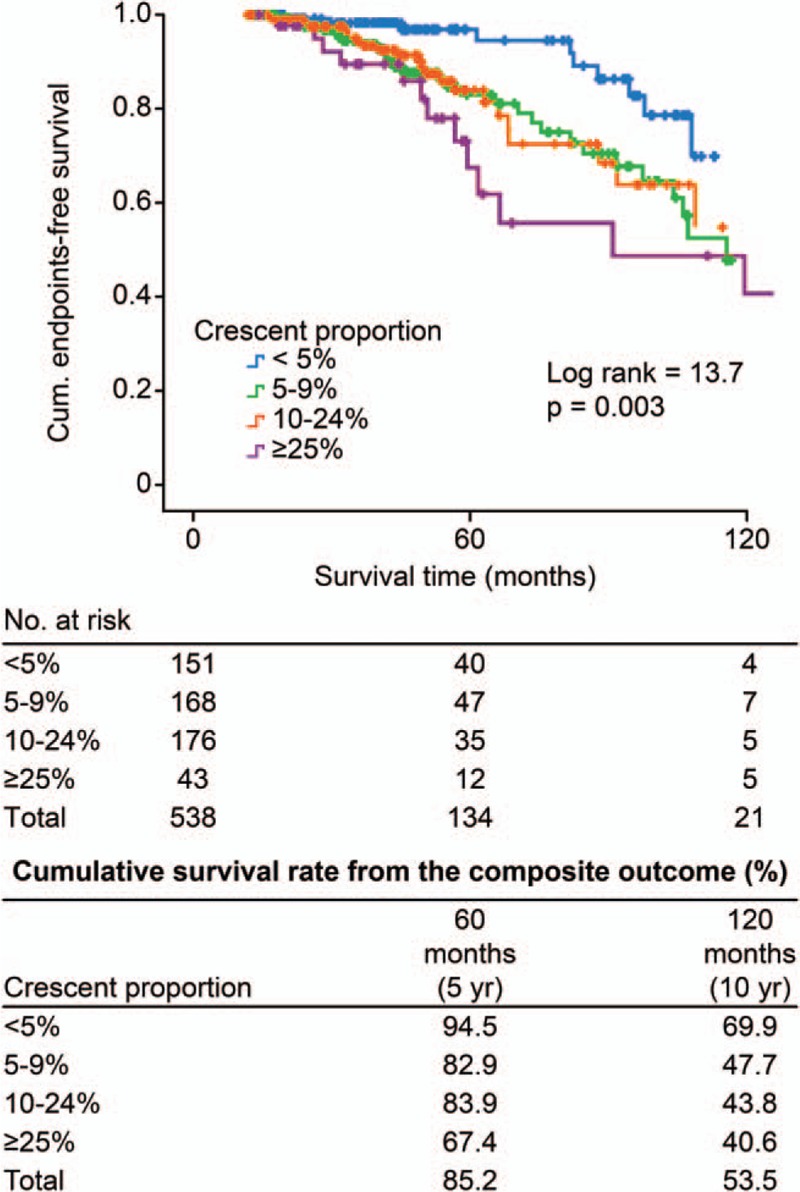
Endpoints-free survival of IgAN patients with different proportions of crescents. IgAN = IgA nephropathy.

After a median follow-up period of 51 months (range 12–154 months), 69 patients (12.8%) reached renal outcomes. A total of 10 patients achieved doubling of SCr before developing renal failure, and 59 patients reached ESRD. Nine patients died (1.7%), including 5 cases with a crescent proportion ≥25%, 3 cases with a crescent proportion ≥10% and <25%, and 1 case with a crescent proportion ≥5% and <10%. There were 6.6%, 16.7%, 14.2%, and 34.9% of patients reaching the composite endpoint in the subgroups of <5%, 5–9%, 10–24%, and ≥25%, respectively. Renal outcome-free survival rates were comparable between the 4 subgroups; the 5-year cumulative renal survival rates were 94.5%, 82.9%, 84.6%, and 80.7%, respectively, and the 10-year cumulative rates were 69.9%, 52.4%, 59.5%, and 48.6%, respectively (log rank test χ^2^ = 6.84, *P* = 0.08) (Fig. 2). When we incorporated death with renal outcome as a composite endpoint, Kaplan–Meier survival curves showed that there were 69.9%, 47.7%, 43.8%, and 40.6% of patients in the 4 subgroups who developed the endpoint events (log rank test χ^2^ = 13.7, *P* = 0.003) (Fig. 3). Univariate Cox regression analyses (Table 2) revealed that eGFR, hypertension, proteinuria, anemia, hyperuricemia, hypercholesterolemia, hypertriglyceridemia, crescentic proportion (each 5% increase), diffuse mesangial hypercellularity, segmental sclerosis, and tubular atrophy were related to the development of adverse outcomes. In a multivariate model adjusting for eGFR, hypertension, proteinuria, and the Oxford-MEST classification, the crescentic proportion (each increase by 5% [log-transformed]: HR = 1.51, 95% CI 1.08–2.11, *P* = 0.02), eGFR (each increase by 1 mL/min per 1.73 m^2^ [log-transformed]: HR = 0.33, 95% CI 0.18–0.58, *P*< 0.001), hypertension (HR = 1.95, 95% CI 1.11–3.44, *P* = 0.02), proteinuria (each increase by 1 g/24 h [log-transformed]: HR = 1.99, 95% CI 1.44–2.76, *P* < 0.001], diffuse mesangial hypercellularity (HR = 2.63, 95% CI 1.48–4.66, *P* = 0.001) and segmental glomerulosclerosis (HR = 2.60, 95% CI 1.55–4.37, *P* < 0.001) served as independent predictors of unfavorable outcomes, that is, doubling of serum creatinine, ESRD, and death.

**Table 2:**
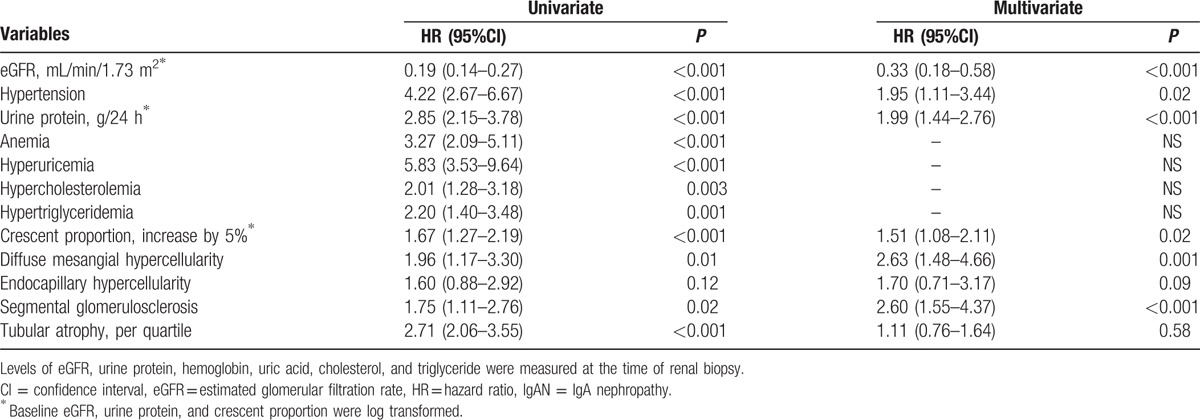
Cox survival analysis: risk factors associated with unfavorable outcomes in crescent-featured IgAN patients.

## 4 Discussion

To the best of our knowledge, the current cohort represents the largest sample size employed to date to investigate the effect of different degrees of crescents on the clinical outcomes of IgAN patients. As previously reported,^[5,11,15]^ we observed that a growing proportion of crescents paralleled a diminishing baseline eGFR and increasing urine protein excretion. In the present study, an increase in the crescent proportion was demonstrated to be one of the determinants of SCr doubling, ESRD, or death after adjusting for traditional risk factors including eGFR, hypertension, and urine protein, together with the following 4 Oxford pathological parameters: mesangial hypercellularity (M), endocapillary hypercellularity (E), segmental glomerulosclerosis (S), and tubular atrophy/interstitial fibrosis (T). Our findings support the explicit association of the crescent proportion with IgAN prognosis.

Clinical characteristics such as initial renal dysfunction (eGFR < 60 mL/min per 1.73 m^2^), proteinuria ≥1 g/d, and hypertension have been identified as predictors of unfavorable prognosis in IgAN.^[16–18]^ A major advance in predicting the risk of progression in IgAN came with the Oxford-MEST pathology classification.^[14,19]^ Although the value of crescents was not confirmed in the original and subsequent validation cohort studies,^[2,9,20]^ a growing number of controversial findings have been reported in recent years.^[1,3,21]^ Accordingly, we further analyzed whether crescents overall (present versus absent), as well as small numbers of crescents (e.g., in <5% of glomeruli and in 5–9% of glomeruli), independently impacted outcomes in patients with IgA nephropathy, utilizing data collected from January 2006 to December 2011 (See Supplemental Figure 1). Compared to the noncrescent counterparts, crescents-featured patients manifested with a reduced eGFR, more frequent gross hematuria, increased amount of urine protein excretion, and presented more serious pathological lesions (see Supplemental Table 1). Cox regression model adjusting for Oxford-MEST pathological parameters showed that crescents were significantly associated with poor outcomes (HR = 1.60, 95%CI 1.07–2.39, *P* = 0.023), whereas the predictive significance was attenuated (HR = 1.11, 95%CI .73–1.71, *P* = 0.624) after adjusting for traditional clinical risk factors consisting of eGFR, hypertension, and proteinuria (See Supplemental Table 2). The conflicting results may be attributed to different inclusion criteria, discrepant histological assessment, sample size, and inconsistent definition of clinical outcomes (doubling of SCr/ 50% decline of eGFR/ ESRD/ death).

It is well accepted that crescents involving >50% of glomeruli in IgAN portend a rapid deterioration of renal function. With regard to the formation of small and focal crescents, their prognostic significance has mostly been demonstrated using an unadjusted model, and the thresholds of crescent proportion for predicting adverse outcomes of IgAN patients in previous studies were 10%, 25%, and 30%.^[1,10,22,23]^ Abe et al^[24]^ reported an increased risk of progression to ESRD with a growing proportion of crescents from the initial biopsy; the 10-year survival rate of IgAN patients with 25% to 50% crescents was approximately 80%. Nearly 50% of crescentic IgAN patients developed ESRD within 3 years, and the 10-year survival rate was only 20%. Tumlin et al^[25]^ retrospectively analyzed a series of 12 IgAN patients with at least 10% crescents or endocapillary proliferation and found that, if untreated, the incidence of ESRD reached 40% within 3 years. Our data showed that crescents affecting 5% to 9% of glomeruli carried an independent risk of unfavorable prognosis (HR = 1.76, 95%CI 1.07–2.90, *P* = 0.03) adjusting for traditional clinical risk factors and Oxford–MEST parameters, whereas IgAN patients presenting crescents <5% glomeruli had comparable risk of progression to those absent of such pathological lesion (See Supplemental Tables 3 and 4). Kaplan–Meier survival analysis (See Supplemental Figure 2) revealed that the 5-year cumulative endpoint events-free survival rate for patients without crescents and with crescents in <5%, 5% to 9% of glomeruli were 91.0%, 90.4%, and 73.8%, respectively (log rank = 11.9, *P* < 0.001). It is also worth mentioning that when we integrated death with renal outcome as a composite endpoint, we discovered obvious differences between the 4 groups in our study (*P* = 0.003), and each 5% increase in crescent proportion independently carried an additional risk of developing poor outcomes. Therefore, it is reasonable to speculate that the underlying relationship between the crescent proportion and renal outcome may be more explicit if more ESRD patients could be caught “on the spot” before death.

Of note, our cohort did not depict the “natural history” of IgAN patients with crescents, but rather the “current history” of this characterized population, as a large majority of patients were treated with RAS inhibitors, and approximately 40% of them were treated with steroid/immunosuppressive therapy. Therefore, treatment during hospitalization needs to be taken into consideration as one of the confounding factors when assessing long-term prognosis. Although the 2012 KDIGO Guidelines for Glomerulonephritis recommend steroid and cyclophosphamide therapy for crescentic IgAN, there have been no well-established recommendations for the evaluation and treatment of IgAN with a small number of crescents. Several studies have shown that active lesions, that is, those with cellular/fibrocellular crescents, endocapillary hypercellularity, and fibrinoid necrosis, demonstrate a good response to immunosuppressive treatment.^[25–27]^ One recent retrospective study evaluating the reversal of active renal pathological lesions after immunosuppressive treatment and its association with IgAN outcomes^[28]^ showed that cellular/fibrocellular crescents were significantly reduced after immunosuppressive therapy (85.0 vs 25.0%, *P* < 0.001), accompanied by clinical remission of proteinuria and hematuria. None but chronic lesions such as tubular atrophy and interstitial fibrosis were confirmed to adversely affect renal outcome. Empirical treatment at our center tends to include immunosuppressive therapy consisting of corticosteroids (regularly orally administered prednisone or intravenous-pulsed methylprednisolone), as more glomeruli are involved in cases with cellular/fibrocellular crescents. Effective therapeutic intervention and the reversal of active extracapillary proliferation may also explain the inconspicuous disparity in renal outcomes, as a greater percentage of patients in higher crescent proportion received immunosuppressive treatment.

Our results should be prudently interpreted in light of the limitations of this study. First, approximately one-quarter of the patients were unavailable to be contacted; hence, a slight selection bias was inevitable in this retrospective single-center study. Second, therapeutic regimes were flexible according to physicians’ clinical decision making, and immunosuppression, in particular, was not standardized, so that such unadjusted confounding impairs data interpretation. Third, this is a retrospective cohort study with patients enrolled from a single hospital; hence, whether our results apply to IgAN patients having distinctive demographic features is uncertain. The influence of crescents at different proportions on renal outcomes in IgAN should be further evaluated in a long-term prospective study.

## 5 Conclusion

In conclusion, the present study showed that an increasing crescent proportion in IgAN was independently associated with unfavorable outcomes, even after adjusting for clinical factors and Oxford-MEST pathological parameters. The prognostic value of the crescent proportion remains to be further consolidated considering the influence of immunosuppression and needs to be assessed in larger prospective studies.

## Supplementary Material

Supplemental Digital Content
