# Supplementary material for: Clinical outcomes of IgA nephropathy patients with different proportions of crescents
Source: Medicine (Baltimore). 2017 Mar 24;96(11):e6190. doi: 10.1097/MD.0000000000006190 (PMC5369886; doi:10.1097/MD.0000000000006190)
Supplement: Supplemental Digital Content [file medi-96-e6190-s001.doc]

***Supplemental Content***

**Clinical Outcomes of IgA Nephropathy Patients with Different Proportions of Crescents**

*An Observational Cohort Study(STROBE Complaint)*

*Wang Zhang^1^PhD, Qian Zhou^1^ MSc, Lingyao Hong^1^ MSc, Wenfang Chen ^2^ PhD, Shicong Yang ^2^ PhD，Qiongqiong Yang^1^ MD, PhD, Wei Chen^1*^ MD, PhD, Xueqing Yu^1^ MD, PhD.*


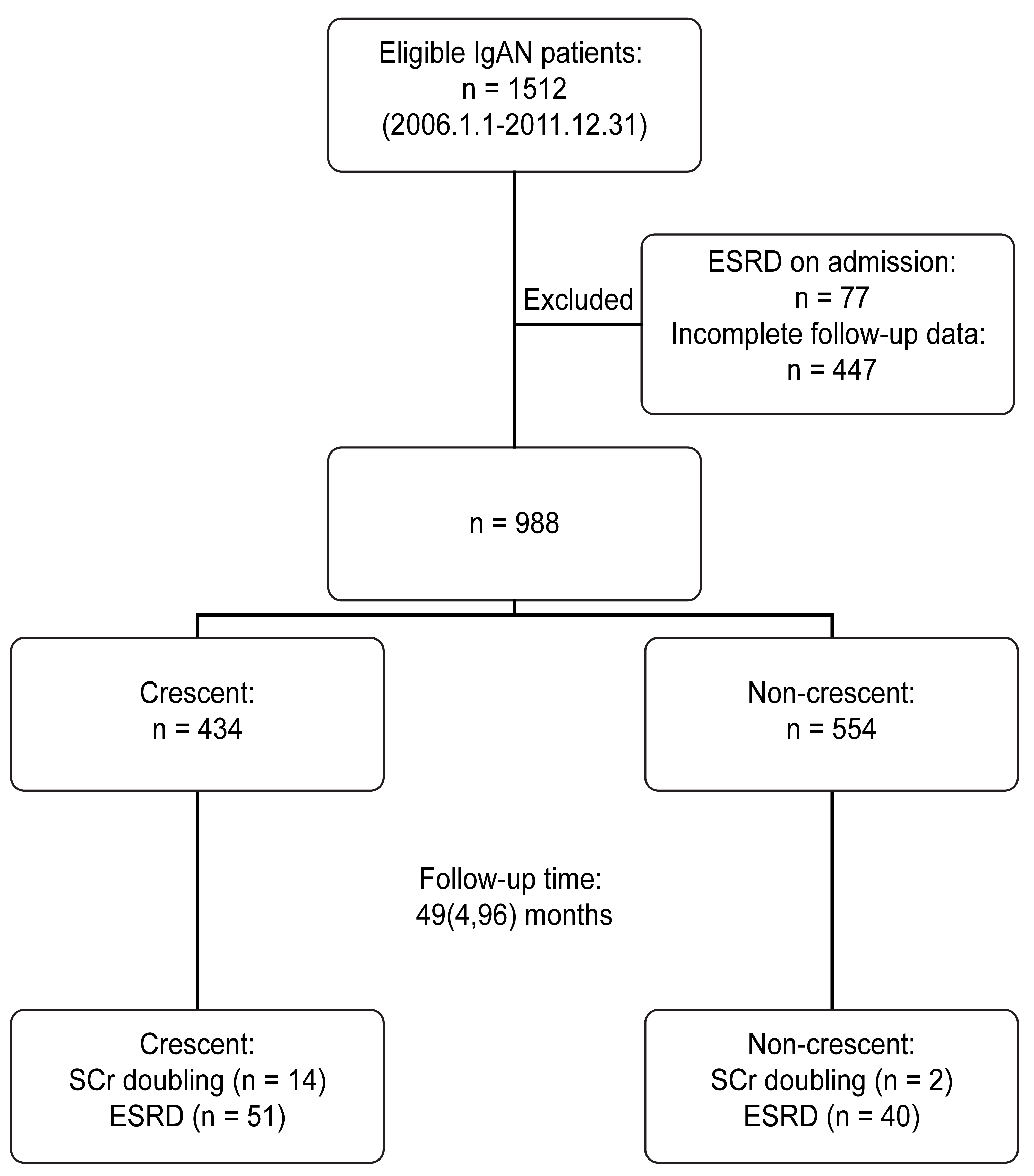


**Supplemental Figure 1. Flow chart of enrollment.**


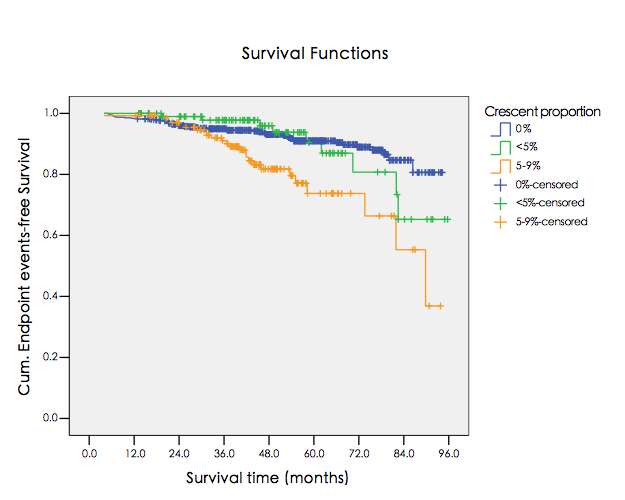
__

Log rank=17.9

P<0.001

| No. of risk |  | 3yr | 5yr |
| --- | --- | --- | --- |
| 0% | 554 | 417 | 194 |
| <5% | 107 | 74 | 26 |
| 5-9% | 138 | 97 | 21 |
| Total | 799 | 588 | 241 |

Cumulative survival rate from the composite outcome (%)

|  | 3 yr | 5yr |
| --- | --- | --- |
| 0% | 95.0 | 91.0 |
| <5% | 97.8 | 90.4 |
| 5-9% | 91.1 | 73.8 |
| Total | 94.7 | 88.5 |

**Supplemental Figure 2. Endpoints-free survival of IgAN patients with a small number of crescents.**

**Supplemental Table 1. Comparisons of baseline clinicopathological characteristics between IgAN patients with and without crescents.**

| **Variables** | **All** | **Non-crescent** | **Crescent** | **p-value** |
| --- | --- | --- | --- | --- |
|  | **n=988** | **n=554** | **n=434** |  |
| Gender [n(%)]  Male  Female | 417(42.2)  571(57.8) | 222(40.1)  332(59.9) | 195(44.9)  239(55.1) | .13 |
| Age^a^[yr] | 32(26,39) | 33(27,40) | 30(25,38) | .001 |
| eGFR^a^[ml/min/1.73 m^2^] | 96(63,124) | 105(69,128) | 88(57,116) | < .001 |
| Gross hematuria^a^ [n(%)] | 280(28.3) | 138(24.9) | 142(32.7) | .007 |
| SBP^a^ [mmHg]  DBP[mmHg] | 123(112,136)  79(70,88) | 122(111,135)  79(70,88) | 125(113,139)  78(70,87) | .03  .20 |
| Hb^a^[g/L] | 128(115,140) | 128(116,142) | 125(113,138) | .01 |
| UA^a^[umol/L] | 349(270,431) | 330(260,419) | 366(294,458) | < .001 |
| ALB^a^[g/L] | 40(37,43) | 40(37,43) | 39(35,42) | < .001 |
| Urine protein^a^[g/24h] | 0.59(0.30,1.20) | 0.51(0.25,1.03) | 0.74 (0.39,1.52) | < .001 |
| Urine RBC^a^[n(%)]  +  ++  +++  ++++ | 267(27.0)  209(21.2)  135(13.7)  51(5.2) | 156(28.2)  95(17.1)  59(10.6)  28(5.1) | 111(25.6)  114(26.3)  76(17.5)  23(5.3) | < .001 |
| Global glomerulosclerosis^a^[%] | 11(0,31) | 9(0,29) | 13(4,31) | .004 |
| Segmental glomerulosclerosis^a^[%] | 0(0,8) | 0(0,6) | 3(0,9) | < .001 |
| Mesangial hypercellularity^a^[n(%)]  Focal  Diffuse | 381(38.6)  516(52.2) | 229(41.3)  262(47.3) | 152(35.0)  254(58.5) | .001 |
| Endocapillary hypercellularity^a^[%]  Focal  Diffuse | 189(19.1)  11(1.1) | 71(12.8)  1(0.2) | 118(27.2)  10(2.3) | < .001 |
| Fibrinoid necrosis^a^ [n(%)] | 89(9.0) | 23(4.2) | 66(15.2) | < .001 |
| Interstitial inflammation^a^[n%]  Mild  Moderate  Severe | 586(57.5)  158(15.5)  20(2.0) | 306(52.9)  81(14.0)  8(1.4) | 280(63.3)  77(17.4)  10(2.3) | < .001 |
| Interstitial fibrosis^a^[n(%)]  Mild  Moderate  Severe | 385(39.0)  181(18.3)  25(2.5) | 187(33.8)  90(16.2)  13(2.3) | 198(45.6)  91(21.0)  12(2.8) | < .001 |
| Tubular atrophy^a^[n(%)]  Mild  Moderate  Severe | 499(50.5)  227(23.0)  21(2.1) | 266(48.0)  109(19.7)  11(1.9) | 233(53.7)  118(27.2)  10(2.3) | < .001 |
| Oral prednisone^a^[n(%)] | 294(29.9) | 123(22.3) | 171(39.5) | < .001 |
| MP iv. Pulse^a^[n(%)] | 71(7.2) | 13(2.3) | 58(13.4) | < .001 |

^a^p<0.05: non-crescent vs. crescent

eGFR= estimated glomerular filtration rate, Hb= haemoglobin, UA= uric acid, ALB= albumin, RBC= red blood cell, RAS= rennin angiotensin system, MP= methylprednisolone.

Normally distributed quantitative variables were expressed as the mean± SD, and nonparametric data were expressed as the median (interquartile range, IQR). Categorical variables were expressed in frequencies (percentages).

**Supplemental Table 2. Cox survival analysis: predictive value of crescent on clinical outcomes in IgAN.**

|  | **HR (95%CI)** | **p-value** |  |
| --- | --- | --- | --- |
| **Model 1** |  |  |  |
| Crescent | 2.36 (1.59-3.49) | < .001 |  |
| **Model 2** |  |  |  |
| Crescent | 1.60 (1.07-2.39) | .023 |  |
| Segmental glomerulosclerosis | 2.87 (1.85-4.45) | < .001 |  |
| Tubular atrophy（per quartile） | 5.47 (4.09-7.32) | < .001 |  |
| **Model 3** | | | |
| Crescent | 1.11 (0.73-1.71) | .624 |  |
| Baseline eGFR^*^ | 0.17 (0.12-0.24) | < .001 |  |
| Urine protein^*^ | 1.54 (1.20-1.98) | .001 |  |
| Segmental glomerulosclerosis | 2.81 (1.77-4.47) | < .001 |  |
| Tubular atrophy（per quartile） | 2.13 (1.48-3.08) | < .001 |  |

HR= hazard ratio

^*^ Baseline eGFR and urine protein results were log transformed

Model 1: unadjusted

Model 2: adjusted for mesangial hypercellularity(M), endocapillary hypercellularity(E), segmental sclerosis(S), tubular atrophy(T)

Model 3: adjusted for age, sex, baseline eGFR, hypertention, urine protein and MEST.

**Supplemental Table 3. Baseline clinicopathological characteristics of IgAN patients with no and a small number of crescents.**

| Variables | **Group 1**  **n=554** | **Group 2**  **n=107** | **Group 3**  **n=138** |
| --- | --- | --- | --- |
| Gender ^a^ [n (%)] |  |  |  |
| Male | 222 (40.1) | 55 (51.4) | 54 (39.1) |
| Female | 332 (59.9) | 52 (48.6) | 84 (60.9) |
| Age [yr] | 33 (27,40) | 33 (24,39) | 31 (27,39) |
| eGFR ^b^ [ml/min/1.73 m^2^] | 99±46 | 101±41 | 88±42 |
| Hypertension [n (%)] | 185 (33.4) | 37 (34.6) | 47 (34.1) |
| Gross haematuria [n (%)] | 138 (24.9) | 39 (36.4) | 44 (31.9) |
| Hb ^b^ [g/L] | 128±19 | 123±19 | 124±17 |
| UA ^b^ [mol/L] | 330 (260,419) | 348(267,422) | 365(279,430) |
| ALB[g/L] | 40 (37,43) | 41 (38, 43) | 39 (36, 43) |
| Urine protein ^b^[g/24 h] | 0.5 (0.3,1.0) | 0.5 (0.3, 1.0) | 0.7 (0.4, 1.1) |
| Urine RBC ^b^ |  |  |  |
| + | 156 (28.2) | 27 (25.2) | 36 (26.1) |
| ++ | 95 (17.1) | 27 (25.2) | 35 (25.4) |
| +++ | 59 (10.6) | 16 (15.0) | 28 (20.3) |
| ++++ | 28 (5.1) | 2 (1.9) | 9 (6.5) |
| Global glomerulosclerosis ^b^ [%] | 9 (0,29) | 8 (0, 23) | 15 (5, 33) |
| Segmental glomerulosclerosis ^a,b^ [%] | 0 (0,6) | 3 (0, 6) | 3 (0, 9) |
| Diffuse mesangial hypercellularity ^b^ [n (%)] | 262 (47.3) | 61 (57.0) | 79 (57.2) |
| Endocapillary hypercellularity ^b^ [n (%)] | 72 (13.0) | 22 (20.6) | 35 (25.4) |
| Interstitial inflammation ^b^ [n (%)] |  |  |  |
| Mild | 306 (52.9) | 64 (59.8) | 95 (68.8) |
| Moderate | 81 (14.0) | 11 (10.3) | 18 (13.0) |
| Severe | 8 (1.4) | 1(0.9) | 4 (2.9) |
| Interstitial fibrosis ^b^ [n (%)] |  |  |  |
| Mild | 187(33.8) | 49 (45.8) | 69 (50.0) |
| Moderate | 90(16.2) | 13 (12.1) | 28 (20.3) |
| Severe | 13(2.3) | 1 (0.9) | 3 (2.2) |
| Tubular atrophy ^b^ [n (%)] |  |  |  |
| Mild | 266(48.0) | 63 (58.9) | 76 (55.1) |
| Moderate | 109(19.7) | 17 (15.9) | 37 (26.8) |
| Severe | 11(1.9) | 1 (0.9) | 3 (2.2) |

Group1: no crescent; Group2: crescent proportion>0% and <5%; Group3: crescent proportion≥5% and <10%.

^a^ p<0.05: Group2 vs. Group1; ^b^ p<0.05: Group3 vs. Group1.

eGFR= estimated glomerular filtration rate, Hb= haemoglobin, UA= uric acid, ALB= albumin, RBC= red blood cell, RAS= rennin angiotensin system, MP= methylprednisolone.

Normally distributed quantitative variables were expressed as the mean± SD, and nonparametric data were expressed as the median (interquartile range, IQR). Categorical variables were expressed in frequencies (percentages).

**Supplemental Table 4. Impact of crescents in <5% and 5%-9% of glomeruli on clinical outcomes of IgAN.**

| **Crescent proportion** |  | **Model 1** | |  | **Model 2** | |  | **Model 3** | |
| --- | --- | --- | --- | --- | --- | --- | --- | --- | --- |
|  |  | **HR (95%CI)** | **p-value** |  | **HR (95%CI)** | **p-value** |  | **HR (95%CI)** | **p-value** |
| **0%** |  | as reference | | | | | | | |
| **<5%** |  | 1.14  ( .56-2.33) | .72 |  | 1.03  ( .50-2.12) | .94 |  | .96  (0.45-2.05) | .91 |
| **5%-9%** |  | 2.86  (1.75-4.66) | <.001 |  | 2.14  (1.31-3.50) | .003 |  | 1.76  (1.07-2.90) | .03 |

HR= hazard ratio

Model 1: unadjusted

Model 2: adjusted for mesangial hypercellularity(M), endocapillary hypercellularity(E), segmental sclerosis(S), tubular atrophy(T)

Model 3: adjusted for age, sex, baseline eGFR, hypertention, urine protein and MEST.
